# Susceptibility to Aminoglycosides and Distribution of *aph* and *aac(3)-XI* Genes among *Corynebacterium striatum* Clinical Isolates

**DOI:** 10.1371/journal.pone.0167856

**Published:** 2016-12-09

**Authors:** Jesús Navas, Marta Fernández-Martínez, Carlos Salas, María Eliecer Cano, Luis Martínez-Martínez

**Affiliations:** 1 Departamento de Biología Molecular, Facultad de Medicina, Universidad de Cantabria, Herrera Oria s/n, Santander, Spain; 2 Servicio de Microbiología, Hospital Universitario Marqués de Valdecilla-IDIVAL, Avda. de Valdecilla s/n, Santander, Spain; Institut National de la Recherche Agronomique, FRANCE

## Abstract

*Corynebacterium striatum* is an opportunistic pathogen, often multidrug-resistant, which has been associated with serious infections in humans. Aminoglycosides are second-line or complementary antibiotics used for the treatment of *Corynebacterium* infections. We investigated the susceptibility to six aminoglycosides and the molecular mechanisms involved in aminoglycoside resistance in a collection of 64 *Corynebacterium striatum* isolated in our laboratory during the period 2005–2009. Antimicrobial susceptibility was determined using E-test. The mechanisms of aminoglycoside resistance were investigated by PCR and sequencing. The 64 *C*. *striatum* were assessed for the possibility of clonal spreading by Pulsed-field Gel Electrophoresis (PFGE). Netilmicin and amikacin were active against the 64 *C*. *striatum* isolates (MICs_90_ = 0.38 and 0.5 mg/L, respectively). Twenty-seven of the 64 *C*. *striatum* strains showed a MIC_90_ for kanamycin > 256 mg/L, and 26 out the 27 were positive for the *aph(3’)-Ic* gene. Thirty-six out of our 64 *C*. *striatum* were streptomycin resistant, and 23 out of the 36 carried both the *aph(3”)-Ib* and *aph(6)-Id* genes. The gene *aac(3)-XI* encoding a new aminoglycoside 3-N acetyl transferase from *C*. *striatum* was present in 44 out of the 64 isolates, all of them showing MICs of gentamicin and tobramycin > 1 mg/L. CS4933, a *C*. *striatum* showing very low susceptibility to kanamycin and streptomycin, contains an aminoglycoside resistance region that includes the *aph(3’)-Ic* gene, and the tandem of genes *aph(3”)-Ib* and *aph(6)-Id*. Forty-six major PFGE types were identified among the 64 *C*. *striatum* isolates, indicating that they were mainly not clonal. Our results showed that the 64 clinical *C*. *striatum* were highly resistant to aminoglycosides and mostly unrelated.

## Introduction

The genus *Corynebacterium* consists of more than 100 species (https://www.dsmz.de/bacterial-diversity/prokaryotic-nomenclature-up-to-date, accessed November 18, 2016). *Corynebacterium* spp. are widely distributed in the environment. Pathogenic *Corynebacterium* species include *Corynebacterium diphtheriae* and nondiphtheroid *Corynebacterium*. Nondiphtheroid corynebacteria are part of the normal skin and mucous membranes microbiota of humans. They have traditionally been considered as sample contaminants when isolated from clinical stuffs. However, nondiphtheroid *Corynebacterium* are actually recognized as causing opportunistic disease [1], particularly in specific circumstances, such as repeated exposure to broad-spectrum antibiotics, after the use of invasive medical procedures or in patients who have suffered long-term hospitalization [2,3,4]. *Corynebacterium striatum* has been implicated sporadically as etiological agent of a wide variety of infections, such as bacteraemia associated with central venous catheters, arthritis, vertebral osteomyelitis, septicaemia, meningitis, endocarditis, breast abscess, peritonitis, wound infections, and prosthetic joint infections [5], in both immunocompetent and immunocompromised patients [3]. In two *C*. *striatum* outbreaks, patient-to-patient transmission of a single *C*. *striatum* strain was reported [6,7].

Hospitals are “antibiotic-rich” environments, creating a selective pressure on the survival of resistant bacteria by which resistance genes can be transferred to susceptible bacteria, spreading multi-resistance. It has been suggested that this selective pressure favours the overgrowth of *C*. *striatum* as a secondary colonizer in immunocompromised hosts [6], so that *C*. *striatum* is considered a multi-resistant nosocomial pathogen [5]. In this context, the emergence of multidrug resistant strains is of particular concern, because the therapeutic options for the management of severely ill patients can be limited, increasing the reliance on vancomycin, which is still active against practically all clinical isolates of the *C*. *striatum*. Therapeutic efficacy of vancomycin can be improved if it is combined with a second antibiotic, most often an aminoglycoside. Thus, several cases of endocarditis due to *C*. *striatum* were successfully treated with gentamicin combined with vancomycin [8,9]. Therefore, aminoglycosides are considered as complementary antibiotics for the treatment of *C*. *striatum* infections.

There is a need for more data from clinical isolates to evaluate the risk of emergence of antimicrobial-resistant *C*. *striatum* strains and to track the transfer of resistance genes. In this respect, we aimed to investigate the susceptibility to six aminoglycosides of 64 clinical isolates of *C*. *striatum* from our sanitary area and characterized the molecular mechanisms of resistance. The 64 *C*. *striatum* were typed by means of PFGE to inquire about their clonal relationships and possible spread mechanisms.

## Material and Methods

### Bacterial strains and growth conditions

Sixty-four *C*. *striatum* obtained from clinical samples of 63 patients collected during the period 2005–2009 at the Clinical Microbiology laboratory of the Marqués de Valdecilla University Hospital, Santander, Spain, were included in this study. Only in one case, two isolates from the same patient were included (*C*. *striatum* CS4108 and CS4489), since they showed different PFGE patterns and MICs. The 64 *C*. *striatum* were isolated from surgical wound exudates (32), non-surgical wound exudates (22, of which 7 were from diabetic foot lesions), blood (3), abdominal drainage (1), peritoneal ascitic fluid (2), sputum (2), gluteus abscess (1), and bronchoalveolar lavage fluid (1). They were grown on blood agar plates at 37°C. All isolates were identified as putative *C*. *striatum* using API CORYNE V2 strips (bioMérieux, Marcy l’Etoile, France). *C*. *striatum* was differentiated from *C*. *amycolatum* using additional phenotypic tests (tyrosine hydrolysis, N-acetylglucosamine assimilation and phenylacetic acid assimilation). Identification was confirmed by MALDI-TOF, using the Vitek-MSTM (bioMérieux) system, according to manufacturer’s instructions.

### Susceptibility to aminoglycosides

The aminoglycosides used in this study were chosen because either they are frequently used in clinical practice (amikacin, gentamicin, netilmicin, and tobramycin) or are good markers of resistance mechanisms (kanamycin and streptomycin). MICs were determined using Etest® (bioMérieux) on Mueller-Hinton agar (Pronadisa, Madrid, Spain), following the instructions of the manufacturer. Clinical categories were established according to breakpoints defined by the European Committee on Antimicrobial Susceptibility Testing (EUCAST) [10]. Since EUCAST has only defined breakpoints for gentamicin, MIC values of amikacin, kanamycin, netilmicin and tobramycin were interpreted according to EUCAST criteria for *Staphylococcus* spp. The EUCAST lacks breakpoints of streptomycin for staphylococci and those for enterococci are specified for detecting high-level resistance, which was not the primary objective of this study. For this reason we have considered the MIC breakpoints values proposed by the French Society of Microbiology (http://www.sfmmicrobiologie.org/UserFiles/files/casfm/CASFM2013vjuin.pdf) to define the number of streptomycin sensitive, intermediate and resistant strains. *Staphylococcus aureus* ATCC 29213 and *Enterococcus faecalis* ATCC 29212 were used as control strains for susceptibility testing assays.

### Pulsed-field gel electrophoresis and dendrogram analysis

PFGE was performed with a CHEF-DRIII system (Bio-Rad, Hercules, California, USA). Bacteria were grown in Brain Heart Infusion agar (Pronadisa) with shaking at 37°C for 48-72h. Cultures were adjusted to A_600_ = 2.0, cells from 250 μl were pelleted and re-suspended in 300 μl of TE buffer (10 mM Tris, 1 mM EDTA) containing 2 mg/ml lysozyme. This suspension was incubated at 37°C for 1 h, inverting the tubes every ten minutes. An equal volume of 2% LM agarose (Pronadisa) in TE buffer containing 1% SDS and 0.2 mg/ml proteinase K was added, and plugs were cast with a standard casting tray. After the plugs solidified, they were incubated overnight at 55°C with shaking in 4 ml of TE buffer containing 1% sarcosyl and 0.15mg/ml proteinase K. The plugs were washed eight times with pre-warmed TE buffer and then digested with 30 U of *XbaI* at 37°C overnight. Electrophoresis was performed in a 1% agarose gel at 6 V/cm and 14°C with 0.5xTBE buffer. Pulse times ramped from 0.1 to 5 seconds for 18h. Low range PFGE marker (New England Biolabs, Beverly, USA) was used as the molecular size marker. Cluster analysis was performed with Fingerprinting II v4.5 software (Bio-Rad, Madrid, Spain) by using the Dice similarity coefficient and the unweighted pair Group method with arithmetic means (UPGMA), with 1% of optimization and tolerance. Isolates were classified as indistinguishable if they showed 100% similarity, as closely related subtypes if they showed 95–99% similarity, and as different strains if they showed <95% similarity.

### Search of aminoglycoside resistance genes by PCR

The 64 *C*. *striatum* were screened for the presence of aminoglycoside modifying enzyme (AME) genes common in gram-positive bacteria [*aac(3)-XI*, *aph(2”)-Ia*, *aph(3’)-Ic*, *aph(3’)-IIIa*, *aph(3”)-Ib*, *aph(6)-Id*, *ant(3”)-Ia*, *ant(4’)-Ia*], or *Enterobacteriae* [*ant(2”)-Ia*, *ant(4’)-IIa*, and *aac(6’)-Ib*] by PCR using primers specific for each gene (S1 Table) and by sequencing of the PCR products. As a control, the 24 isolates susceptible to aminoglycosides were also used in the PCR analysis. DNA was extracted using InstaGene matrix (Bio-Rad) according to manufacturer’s instructions. Then, 10 μl of DNA template (10 pg/μl) was added to a reaction mixture containing 4 U of *Taq* DNA polymerase (Bioline, London, United Kingdom), 1.5 mM MgCl_2_, 5 μl of 10× PCR amplification buffer [160 mM (NH_4_)_2_ SO_4_, 670 mM Tris-HCl (pH = 8.8), 0.1% Tween 20], 40 pmol of each primer, 0.2 mM each deoxynucleoside triphosphate (Bioline), and double-distilled water to a final volume of 50 μl. DNA was first denatured at 95°C for 5 min and then subjected to 30 cycles of amplification using a Perkin Elmer model 9600 thermal cycler under the following conditions: denaturation at 95°C for 1 min, annealing for 1 min, and extension at 72°C for 1 min. After the final cycle, the reactions were terminated by an extra run at 72°C for 10 min, and the reaction products were then kept at 4°C until analysis. Amplification products were characterized by electrophoresis in 1.5% agarose using 0.5x TBE running buffer. PCR products were cleaned of amplification primer using the QIAquick PCR Purification kit (Qiagen, Madrid, Spain) following the manufacturer’s instructions. Purified DNA was sequenced with the primers outlined in S1 Table.

### Genome sequencing and analysis

*C*. *striatum* CS4933 whole DNA was prepared from cultures grown on blood agar plates overnight. Bacterial cells were mixed with 2 ml of distilled water poured on the plate, transferred to a 2-ml Eppendorff tube, collected by centrifugation at 10,000g for 4 min, re-suspended in 0.25 ml of Tris-EDTA buffer containing 20 mg of lysozyme/ml and 50 mg of proteinase K/ml, and incubated at 37°C for 1 h. Bacterial cells were then lysed by the addition of 0.25 ml of 0.1 M Tris containing 1% sodium dodecyl sulfate (SDS) and 400 μg/ml of proteinase K and incubated at 55°C for 1 h. The lysate was mixed with 0.1 ml of 5 M NaCl and 100 μl of cetyltrimethylammonium bromide-NaCl and incubated at 65°C for 10 min. DNA was then extracted with chloroform-isoamyl alcohol and phenol-chloroform, precipitated with isopropanol, and re-suspended gently in distilled water. For genomic sequencing genomic libraries were prepared using the Illumina TruSeq DNA Sample Prep Kit and size-selected to a 400 bp mean insert size using Sage Pippin Blue. Final libraries were sequenced on a MiSeq platform using 250 base paired-end reads (v2 chemistry). Contigs were assembled with the MacVector tool (http://macvector.com), and analysed using the NCBI ORF finder (www.ncbi.nlm.nih.gov/gorf) and BLAST (www.ncbi.nlm.nih.gov/BLAST) tools. The sequence of the *C*. *striatum* CS4933 region including *aph(3’)-Ic*, *aph(3”)-Ib* and *aph(6)-Id* genes has been deposited in GenBank under accession number KP119857.

## Results and Discussion

### Susceptibility of *C*. *striatum* to aminoglycosides

According to the breakpoints previously indicated, 52 of the 64 *C*. *striatum* included in this study were resistant to at least one of the six aminoglycosides, whereas the remaining 12 isolates were aminoglycoside-susceptible. The MIC_50_ and MIC_90_ distributions of the different aminoglycosides for the 64 *C*. *striatum* are presented in Table 1.

**Table 1:** Susceptibility of 64 *Corynebacterium striatum* strains to six aminoglycosides.

					Number of strains	
	Range	MIC_50_(mg/L)	MIC_90_(mg/L)	Resistant	Intermediate	Total susceptible
Amikacin	0.016–256	0.25	0.5	0	0	64
Gentamicin	0.064–1024	2	8	45	0	19
Kanamycin	0.016–256	0.5	>256	27	0	37
Netilmicin	0.016–256	0.094	0.38	0	0	64
Streptomycin	0.064–1024	24	>1024	36	9	19
Tobramycin	0.064–1024	4	16	45	0	19

MIC, minimum inhibitory concentration; MIC_50/90_, MIC that inhibits 50% and 90% of the isolates, respectively.

Considering the MIC_90_, netilmicin was the more active aminoglycoside against the *C*. *striatum* we tested (MIC_90_ = 0.38 mg/L), followed by amikacin (MIC_90_ = 0.5 mg/L). All the isolates were susceptible to these two compounds. Martínez-Martínez et al [11] have previously reported that 27 out of 31 *C*. *striatum* were sensitive to amikacin. In this study, the activity of gentamicin was lower than that of netilmicin and amikacin, with the MIC_90_ of gentamicin (8 mg/L) being 16 times higher than that of amikacin. Forty-five out of 64 isolates were resistant to gentamicin. Other studies reported variable activity of gentamicin against *C*. *striatum*: Campanile et al. [12] found a MIC_90_ of 32 mg/L for 36 *C*. *striatum* isolated in Italy whereas Gómez-Garcés et al. [13] reported a MIC_90_ of 2 mg/L for a collection of 30 Spanish *C*. *striatum*. Tobramycin was scarcely active against our *C*. *striatum* (MIC_90_ = 16 mg/L). The 45 isolates tobramycin-resistant were resistant to gentamicin as well, suggesting a common mechanism involved in resistance to these two compounds. Kanamycin and streptomycin showed poor activity against our strains (MICs_90_ >256 and >1024 mg/L, respectively).

According to EUCAST criteria, six resistance profiles were found among the 52 *C*. *striatum* isolates resistant to at least one of the tested aminoglycosides (Table 2). Forty-four of the 64 isolates were resistant to more than one of the tested aminoglycosides. The most frequently encountered multi resistance phenotype was Kanamycin/Gentamicin/Tobramycin/Streptomycin (n = 24), followed by Gentamicin/Tobramycin (n = 13). Furthermore, eight isolates were resistant to just one of the considered aminoglycosides: seven to streptomycin and one to kanamycin.

**Table 2:** Resistance phenotypes of the 52 *C*. *striatum* strains resistant to aminoglycosides.

Resistance Phenotype	Number of *C*. *striatum* strains
Kan	1
Sm	7
Genta-Tob	13
Genta-Tob-Sm	5
Kan-Genta-Tob	2
Kan-Genta-Tob-Sm	24

Resistance was defined in accordance with European Committee on Antimicrobial Susceptibility Testing (EUCAST) guidelines. Kan: kanamycin; Sm: streptomycin; Genta: gentamicin; Tob: tobramycin.

The data obtained in this study may support the consideration of netilmicin and amikacin in combination with vancomycin for empiric therapy against infections caused by *C*. *striatum*, although more studies are necessary to confirm these results. In any case appropriate antimicrobial therapy against *C*. *striatum* infections must be prescribed on the basis of the data provided by antibiotic susceptibility tests for each strain.

### Detection and mapping of aminoglycoside resistance genes

Inactivation by enzymatic modification is the most prevalent mechanism of resistance of corynebacteria to aminoglycosides in the clinical setting. The *aph(3’)-Ic* gene, encoding an aminoglycoside-O-phosphotransferase conferring resistance to kanamycin, neomycin, paromomycin, ribostamycin and lividomycin, is part of plasmids and transposons and its wide distribution includes *Corynebacterium* spp. [14]. Twenty-six out of the 27 *C*. *striatum* resistant to kanamycin rendered the expected 0.48-kb amplicon when amplified with *aph(3’)-Ic* specific primers. The isolate *C*. *striatum* CS3253 (MIC of kanamycin > 256 mg/L) was not amplified, suggesting that resistance is mediated by allelic variants of the gene or by another mechanism. The 37 *C*. *striatum* susceptible to kanamycin were negative when they were analysed by PCR-*aph(3’)-Ic*. Therefore, in our *C*. *striatum*, there is a correlation between resistance to kanamycin and the presence of the *aph(3’)-Ic* gene. As kanamycin is not prescribed in Spain, the high prevalence of this gene in *C*. *striatum* may be due to its genetic linkage with other resistance genes (see below).

In *Corynebacterium* spp., the main mechanism of streptomycin resistance is related to the presence of the tandem of genes *aph(3”)-Ib* and *aph(6)-Id*, encoding for aminoglycoside-3”-phosphotransferase [APH (3”)-Ib] and aminoglycoside-6-phosphotransferase [APH (6)-Id], respectively [14]. Thirty-six out of our 64 *C*. *striatum* were streptomycin resistant. In 23 streptomycin resistant *C*. *striatum* PCR amplification resulted in the expected *aph(3”)-Ib* (0.55 Kb*)* and *aph(6)-Id* (0.51 Kb) amplicons. However, twelve out of the 24 *C*. *striatum* streptomycin resistant isolates did not give *aph(3”)-Ib* neither *aph(6)-Id* amplicons, suggesting that in these strains streptomycin resistance is mediated by allelic variants of these genes or by another mechanism. One *C*. *striatum* (CS6865) was susceptible to streptomycin (MIC = 1 mg/L) but gave the *aph(3”)-Ib* and *aph(6)-Id* amplicons, which might be due to mutations affecting the *aph(3”)-Ib*-*aph(6)-Id* genes or their promoter region.

Forty-four out of 64 *C*. *striatum* were positive for the gene *aac(3)-XI*, encoding AAC(3)-XI, a new aminoglycoside 3-N acetyl transferase from *C*. *striatum* [15]. AAC (3)-XI acetyl transferase confers resistance to gentamicin, tobramycin, sisomicin, dibekacin and fortimicin. The 44 *C*. *striatum* carrying the *aac(3)-XI* gene showed MICs of both gentamicin and tobramycin > 1 mg/L. Nineteen out the 20 isolates negative for the *aac(3)-XI* gene were susceptible to both gentamicin and tobramycin (MICs < 0.064 mg/L) whereas one isolate showed a MIC = 1.5 mg/L for both compounds. Therefore, there is a good correlation between resistance to these two compounds and the presence of the *aac(3)-XI* gene.

The 44 isolates positive for the gene *aac(3)-XI* have a gentamicin MIC range of 1–16 mg/L, supporting the EUCAST clinical breakpoint of > 1 mg/L. If the gentamicin breakpoint recommended by the CLSI was used (>16 mg/L) [16], most of the isolates positive for the *aac(3)-XI* gene are not considered as resistant. This difference in interpretation between the European and the US recommendations could result in an underestimate of gentamicin resistant *C*. *striatum* if the CLSI breakpoint instead of the EUCAST breakpoint was used, with possible serious consequences for patients.

**Fig 1:**
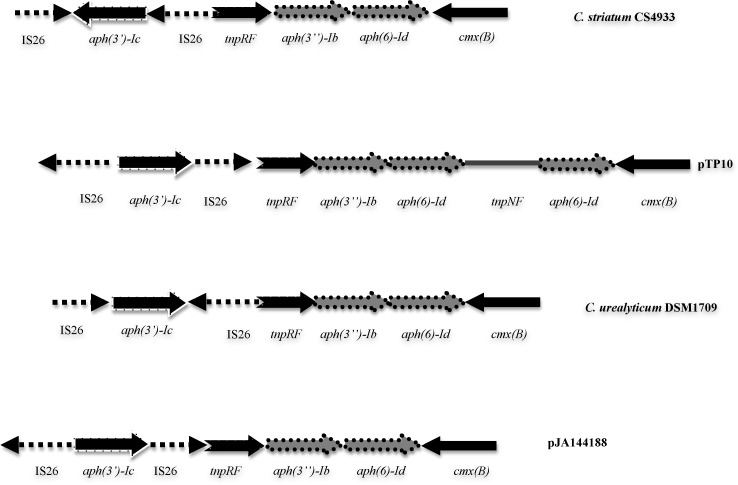
Genomic context of the aminoglycoside resistance genes. Maps of the region including *aph(3’)-Ic*, *aph(3”)-Ib* and *aph(6)-Id* genes of *C*. *striatum* CS4933, *C*. *striatum* plasmid pTP10 [17], *C*. *urealyticum* DSM1709 [18] and *C*. *resistens* plasmid pJA144188 [19].

In order to know the genomic context of the aminoglycoside resistance genes and inquiry about their transfer mechanisms we sequenced the genome of the isolate *C*. *striatum* CS4933, which was chosen for its clinical relevance (it was isolated from soft tissue abscess) and its resistance profile (it was resistant to kanamycin and streptomycin). Annotation of the genome sequence of *C*. *striatum* CS4933 revealed that the *aph(3’)-Ic* gene is flanked by two divergently oriented *IS26*-like elements (Fig 1), forming a new transposon similar to *Tn5715* but with the *aph(3’)-Ic* gene and the *IS26* modules inversely oriented [17]. The *aph(3’)-Ic* gene is part of a larger DNA region containing the *aph(3”)-Ib*—*aph(6)-Id* tandem pair of resistance genes conferring streptomycin resistance. The *aph(3”)-Ib*—*aph(6)-Id* coding regions of *C*. *striatum* CS4933 are located downstream of the resolvase gene from a *Tn5393*-like transposon that is disrupted by the *Tn5715* element containing the *aph(3’)-Ic* gene. Essentially identical aminoglycoside resistance regions were found in the plasmid pTP10 from *C*. *striatum* [17], in the chromosome of *C*. *urealyticum* DSM1709 [18] and in the plasmid pJA144188 of *C*. *resistens* DSM 45100 [19] (Fig 1).

### Molecular epidemiology of the *C*. *striatum* isolates

**Fig 2:**
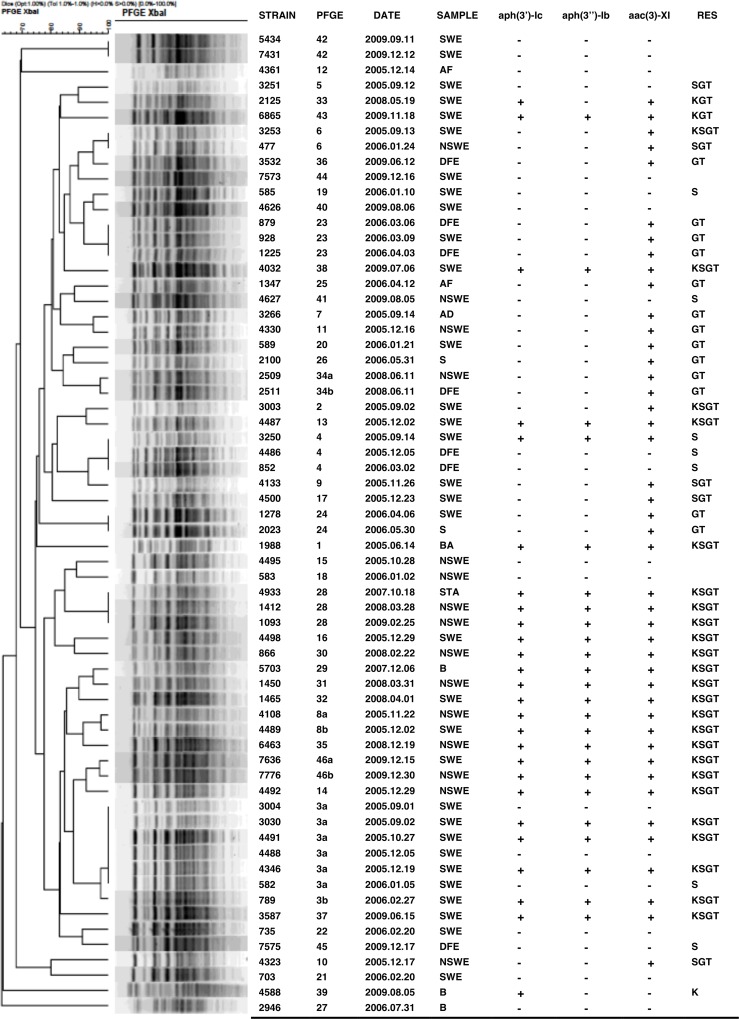
Dendrogram showing PFGE types of the 64 *C*. *striatum*. Sample origin: SWE: surgical wound exudate; AF: ascitic fluid; NSWE: non-surgical wound exudate; DFE: diabetic food exudate; AD: abdominal drainage; S: sputum; BA: bronchial aspirate; STA: soft tissue abscess; B: blood. RES: Resistance profiles; K: kanamycin; S: streptomycin; G: gentamicin; T: tobramycin. G, T in brackets indicates resistance to gentamicin or tobramycin according to European Committee on Antimicrobial Susceptibility Testing (EUCAST) breakpoints.

The PFGE method displayed a high tipability (100% of the isolates were clearly assigned to one pulsotype), and discriminatory power. PFGE delineated the 64 *C*. *striatum* isolates into forty-six distinct PFGE types (Fig 2). On these, PFGE types 3, 8, 34 and 46 could be further classified into 2 subtypes. Most PFGE patterns corresponded to single isolates, whereas one PFGE pattern was observed for 7 isolates, 3 patterns contained three isolates each, and 6 patterns two isolates each. The dendrogram showed a Dice similarity coefficient ranging from 63 to 97% (Fig 2).

Most of the pulsotypes include strains showing the same resistance properties. The 3 strains of the PFGE type 4 were resistant to streptomycin and positive for the genes *aph(3”)-Ib* and *aph(6)-Id*, whereas the 3 strains of the type 23 were sensitive to all aminoglycosides. The 3 strains of the pulsotype 28 were resistant to four aminoglycosides (kanamycin, streptomycin, gentamicin and tobramycin) and contain the *aph(3’)-Ic*, *aph(3”)-Ib*, *aph(6)-Id* and *aac(3)-XI* genes.

High number of pulsotypes in *C*. *striatum* indicated a high genetic diversity in this species. PFGE analysis revealed that our 64 *C*. *striatum* are mostly unrelated, producing sporadic infections.

## Supporting Information

S1 File References for S1 Table. (DOCX)

S1 Table Primers used in the detection of aminoglycoside resistance genes and expected amplicon sizes. (PDF)
