# Supplementary material for: Susceptibility to Aminoglycosides and Distribution of aph and aac(3)-XI Genes among Corynebacterium striatum Clinical Isolates
Source: PLoS One. 2016 Dec 9;11(12):e0167856. doi: 10.1371/journal.pone.0167856 (PMC5148030; doi:10.1371/journal.pone.0167856)
Supplement: S1 File — (DOCX) [file pone.0167856.s001.docx]

**References**

1. Vakulenko SB, Donabedian SM, Voskresenskiy AM, Zervos MJ, Lemer SA, Chow JW. Multiplex PCR for detection of aminoglycoside resistance genes in enterococci. Antimicrob Agents Chemother 2003;47: 1423-6.

2. Miró E, Grünbaum F, Gómez L, Rivera A, Mirelis B, Coll P, Navarro F. Characterization of aminoglycoside-modifying enzymes in *Enterobacteriaceae* clinical strains and characterization of the plasmids implicated in their difusión. Microb Drug Res 2012; 19: 94-9.

3. Udo EE, Dashti AA. Detection of genes encoding aminoglycoside-modifying enzymes in *Staphylococci* by polymerase chain reaction and dot blot hybridization. Int J Antimicrob Agents 2000;13: 273-9.

4. Gebreyes WA, Altier C. Molecular characterization of multidrug-resistant *Salmonella enterica* subsp. *enterica* serovar Typhimurium isolates from swine. J Clin Microbiol. 2002;40: 2813–22.

5. Guerra B, Junke E, Miko A, Helmuth R, Mendoza M. Characterization and localization of drug resistance determinants in multi-drug resistant, integron-carrying *Salmonella enterica* serotype Typhimurium strains. Microb Drug Resist. 2004;10: 83-91.

6. Klingenberg C, Sundsfjord A, Ronnestad A, Mikalsen J, Gaustad P, Flaegstad T. Phenotypic and genotypic aminoglycoside resistance in blood culture isolates of coagulase-negative *Staphylococci* from a single neonatal intensive care unit, 1989-2000. J Antimicrob Chemother 2004;54: 889-96.

7. Park CH, Robicsek A, Jacoby GA, Sahm D, Hooper DC. Prevalence in the United States of *aac(6')-Ib*-cr encoding a ciprofloxacin-modifying enzyme. Antimicrob Agents Chemother 2006;50: 3953-5.

8. Galimand M, Fishovitz J, Lambert T, Barbe V, Zajicek J, Mobashery S, Courvalin P. AAC(3)-XI, a new aminoglycoside 3-N-Acetyltransferase from *Corynebacterium striatum*. Antimicrob Agents Chemother 2015; 59:5647-53.
