# Supplementary material for: Susceptibility to Aminoglycosides and Distribution of aph and aac(3)-XI Genes among Corynebacterium striatum Clinical Isolates
Source: PLoS One. 2016 Dec 9;11(12):e0167856. doi: 10.1371/journal.pone.0167856 (PMC5148030; doi:10.1371/journal.pone.0167856)
Supplement: S1 Table — (PDF) [file pone.0167856.s002.pdf]

GENE	RESISTANCE PHENOTYPE	DNA SEQUENCE (5’-3’)	Annealing temp	PRODUCT (bp)	REFERENCES
aph(2’’)-Ia	Gentamicin, Kan, Tobra (Gram +)	CAGAGCCTTGGGAAGATGAAG<br>CCTCGTGTAATTCATGTTCTGGC	56ºC	348	[1]
aph(3’)-Ic	Kanamycin (*Corynebacterium*)	CGAGCATCAAATGAAACTGC<br>GCGTTGCCAATGATGTTACAG	54ºC	624	[2]
aph(3’)-IIIa	Amikacin, Kanamycin, (Gram+)	GCCGATGTGGATTGCGAAAA<br>GCTTGATCCCCAGTAAGTCA	50ºC	296	[3]
aph(3’’)-Ib	Streptomycin (Gram+)	CTTGGTGATAACGGCAATTC<br>CCAATCGCAGATAGAAGGC	52ºC	548	[4]
aph(6)-Id	Streptomycin (Gram+), (Gram-)	ATCGTCAAGGGATTGAAACC<br>GGATCGTAGAACATATTGGC	50ºC	509	[4]
ant(2’’)-Ia	Gentamicin, Kan, Tobra (Gram -)	ACGCCGTGGGTCGATGTTTGATGT<br>CTTTTCCGCCCCGAGTGAGGTG	60ºC	572	[2]
ant(3’’)-Ia	Streptomycin (Gram+), (Gram-)	GTGGATGGCGGCCTGAAGCC<br>AATGCCCAGTCGGCAGCG	60ºC	526	[5]
ant(4’)-Ia	Amikacin, Kan, Tobra (Gram+)	CAAACTGCTAAATCGGTAGAAGCC<br>GGAAAGTTGACCAGACATTACGAACT	56ºC	294	[6]
ant(4’)-IIa	Gentamicin, Kan, Tobra (Gram-)	ATCGTCTGCGAGAAGCGTAT<br>CGTGTCTTCCACCTCTGGTT	53ºC	356	This work
aac(6’)-Ib	Amikacin, Kan, Netil (Gram -)	TTGCGATGCTCTATGAGTGGCTA<br>CTCGAATGCCTGGCGTGTTT	58ºC	482	[7]
aac(3)-XI	Gentamicin, Tobra (*C. striatum*)	ATGACTACAACCAACGAGATC<br>CTAAAGCTCCCGGATGTAGAG	52ºC	452	[8]
